# The self-renewal of mouse embryonic stem cells is regulated by cell–substratum adhesion and cell spreading^☆^

**DOI:** 10.1016/j.biocel.2013.07.001

**Published:** 2013-11

**Authors:** Patricia Murray, Marina Prewitz, Isabel Hopp, Nicola Wells, Haifei Zhang, Andrew Cooper, Kristina L. Parry, Robert Short, Daniel J. Antoine, David Edgar

**Affiliations:** a Institute of Translational Medicine, The University of Liverpool, Liverpool L69 3GE, UK; b Department of Chemistry, The University of Liverpool, Liverpool L69 7ZD, UK; c University of Sheffield, Department of Material Engineering, Sheffield S1 3JD, UK; d Mawson Institute, The University of South Australia, Mawson Lakes, Adelaide, SA 5095, Australia

**Keywords:** mESC, mouse embryonic stem cell, FAK, focal adhesion kinase, LIF, leukaemia inhibitory factor, ROCK, rho kinase, pPAA, plasma polymerised acrylic acid, PLGA, poly(lactic-co-glycolic acid), Embryonic stem cells, Cell spreading, Biomaterials, E-cadherin, Rho kinase

## Abstract

Mouse embryonic stem cells (mESCs) undergo self-renewal in the presence of the cytokine, leukaemia inhibitory factor (LIF). Following LIF withdrawal, mESCs differentiate, and this is accompanied by an increase in cell–substratum adhesion and cell spreading. The purpose of this study was to investigate the relationship between cell spreading and mESC differentiation. Using E14 and R1 mESC lines, we have restricted cell spreading in the absence of LIF by either culturing mESCs on chemically defined, weakly adhesive biomaterial substrates, or by manipulating the cytoskeleton. We demonstrate that by restricting the degree of spreading by either method, mESCs can be maintained in an undifferentiated and pluripotent state. Under these conditions, self-renewal occurs without the need for LIF and is independent of nuclear translocation of tyrosine-phosphorylated STAT3 or β-catenin, which have previously been implicated in self-renewal. We also demonstrate that the effect of restricted cell spreading on mESC self-renewal is not mediated by increased intercellular adhesion, as evidenced by the observations that inhibition of mESC adhesion using a function blocking anti E-cadherin antibody or siRNA do not promote differentiation. These results show that mESC spreading and differentiation are regulated both by LIF and by cell–substratum adhesion, consistent with the hypothesis that cell spreading is the common intermediate step in the regulation of mESC differentiation by either LIF or cell–substratum adhesion.

## 1 Introduction

Stem cell fate is regulated by soluble factors and interactions involving cell–cell and cell–extracellular matrix (ECM) contacts (Fuchs et al., 2004), as well as by mechanical forces that can regulate stem cell fate through effects on cell shape and spreading (Costa et al., 2012). Numerous studies have demonstrated the important role of cell shape in controlling the differentiation of various types of somatic stem cells including mesenchymal stem cells and epidermal stem cells (McBeath et al., 2004; Gao et al., 2010; Connelly et al., 2010), but less is known about the influence of cell shape or spreading on the fate of embryonic stem cells. It is well-documented that the self-renewal of mouse embryonic stem cells (mESCs) is promoted by the cytokine leukaemia inhibitory factor (LIF) via the JAK-STAT3 signalling pathway (Burdon et al., 2002). Additionally, mechanisms involving the src-related kinase cYes (Anneren et al., 2004) and the Wnt/β-catenin signalling pathway (Sato et al., 2004; Hao et al., 2006) have been implicated in self-renewal, and it has been shown that inhibition of FGF and ERK signalling can promote mESC self-renewal in the absence of LIF (Ying et al., 2008). Prior to down-regulation of the ESC marker, Oct-4, mESCs undergo dramatic shape changes from being tightly packed and rounded, to flattened, spread cells, either following LIF withdrawal (Nichols et al., 2001) or after manipulation of the cYes or Wnt-signalling pathways (Anneren et al., 2004). Furthermore, small-molecule inhibitors that support self-renewal, such as FGF and ERK inhibitors, appear to prevent cell spreading and promote the formation of tightly packed colonies (Ying et al., 2008), which raises the possibility that self-renewal might be promoted by either inhibiting cell spreading and/or promoting cell–cell contact. In support of a role for cell–cell contact in promoting mESC self-renewal, two recent reports have shown that expression of the cell–cell adhesion molecule, E-cadherin, is required for the reprogramming of somatic cells to induced pluripotent stem cells (iPSCs) (Chen et al., 2010; Redmer et al., 2011). However, the existence of E-cadherin-null mESCs that are unable to form tightly packed colonies but nevertheless self-renew, suggests that cell–cell contact is not an absolute requirement for the maintenance of mESC self-renewal (Larue et al., 1996; Soncin et al., 2009).

The importance of cell spreading in the regulation of mESC self-renewal, is highlighted by the fact that if mESCs are cultured on a strongly adhesive surface that forces them to spread, they down-regulate pluripotency markers even when cultured in the presence of LIF (Hayashi et al., 2007; Wells et al., 2009; Hunt et al., 2012). Similar results have been obtained following the induction of mESC cell spreading through the application of local force (Chowdhury et al., 2010; Uda et al., 2011).

The aim of the current study is therefore to investigate the relationship between cell spreading and LIF in the regulation of mESC self-renewal and differentiation, by investigating if differentiation could be inhibited in the absence of LIF by restricting the extent of cell spreading. To this end, cell spreading was regulated either by culturing mESCs on substrates with varying adhesivity, or by manipulating the cytoskeleton. The role of E-cadherin-mediated cell–cell contact, β-catenin localisation and STAT3 signalling in regulating mESC self-renewal under these different conditions was also investigated.

## 2 Materials and methods

### 2.1 Cell culture

E14 and R1 mESC lines were cultured on Nunc^®^ tissue culture dishes coated with either 2.5% (v/v) ESC-tested foetal bovine serum (FBS) (weak adhesion) or 0.1% (w/v) gelatin and 10% (v/v) ESC-tested FBS (PAA laboratories, Yeovil, UK) in serum-free medium comprising Advanced^®^ DMEM (Invitrogen UK Ltd., Paisley, UK) supplemented with 2 mM l-glutamine and 50 μM 2-mercaptoethanol, with or without 1000 U/ml LIF (Millipore, UK) or 10 μM ROCK inhibitor, Y-27632 (Calbiochem). Cells were passaged every 4 days. LIFR^−/−^ cells were a gift from A. Smith and I. Chambers (University of Edinburgh, UK) and were maintained as above except that sIL6 receptor and IL6 were used in place of LIF (Robertson et al., 1993). To promote differentiation, embryoid bodies were produced from ESC single cell suspensions in medium containing 10% (v/v) FBS in non-adherent bacteriological plastic dishes for 4 days and then cultured for 7 days in chamber slides in the absence or presence of 1 μM retinoic acid to promote neural differentiation.

### 2.2 Synthetic culture substrates

RESOMER^®^ polymer LR 708 (poly(lactic-co-glycolic acid) 70:30, Boehringer Ingelheim Bracknell, UK) was dissolved in dichloroethane at a concentration of 3% (w/w) and the polymer substratum fabricated by freeze-drying (Zhang et al., 2005). Prior to culture, the thin dry films were rinsed with PBS and sterilised by soaking in 100% ethanol overnight. Ethanol was removed by washing 3 times in sterile distilled water and once in sterile phosphate buffered saline (PBS). A negatively charged substratum with controlled surface density of carboxylic acid functionality was fabricated by Plasso Technology Ltd. (a spin-out company of The University of Sheffield, UK), via plasma polymerisation of acrylic acid with octadiene on 3.5 cm bacteriological Petri dishes (France et al., 1998). The number of carboxylic acid groups per one hundred carbon atoms was determined to be ∼12% by X-ray photoelectron spectroscopy.

### 2.3 Microscopy

Immunofluorescence was performed as previously described (Smyth et al., 1999). Fixed cells were incubated overnight at 4 °C with primary antibodies to Oct-4, β-catenin, pan-STAT3, pY-STAT3, pan-FAK (all from Santa Cruz, Heidelberg, Germany), Nanog (Abcam, Cambridge, UK), von Willebrand Factor, laminin-1 (all from Sigma, Poole, UK), TUJ1 (R&D Systems Europe, Ltd., Abingdon, UK), alpha-fetoprotein (ICN Biomedicals Ltd., Irvine, UK) or pFAK (Cell Signalling, Beverly, USA). Secondary antibodies were anti-rabbit-488 and -594, anti-mouse IgG2a-594, anti-mouse IgG2b-488 and -594, anti-mouse IgG1-488 (all from Invitrogen). F-actin was visualised using phalloidin labelled with Alexa fluor 594 (Invitrogen). Digital images were acquired using a Leitz DMRB microscope with 20× air and 40× oil objectives with a Sensys^®^ camera and recorded with Metamorph^®^ software. For sub-cellular localisation, samples were observed by confocal microscopy using a Leica TCS-SP2 microscope with 40× oil objective. Images were recorded digitally using Leica software. Quantitative image analysis used 3 × 3 pixel square masks placed at five random points over the membrane, cytoplasm and nucleus for the determination of the intensities. Images for reproduction were prepared with Adobe Photoshop and Illustrator CS-2.

### 2.4 siRNA and RT-PCR

siRNA duplexes (CCGAGAGAGTTACCCTACA) targeted to region 1135-53 of E-cadherin mRNA together with control siRNA duplexes were from Eurogentec Ltd. 50 nM siRNA was introduced using Lipofectamine™ (Invitrogen) according to manufacturer's instructions. RT-PCR or immunofluorescence analysis was performed 24–96 h following transfection. Real-time mRNA quantification was performed with a Rotagene 3000 (Corbett Research Pty., Australia) using Sybr Green JumpStart Taq ReadyMix (Sigma) as described (Murray et al., 2007).

## 3 Results and discussion

**Fig. 1:**
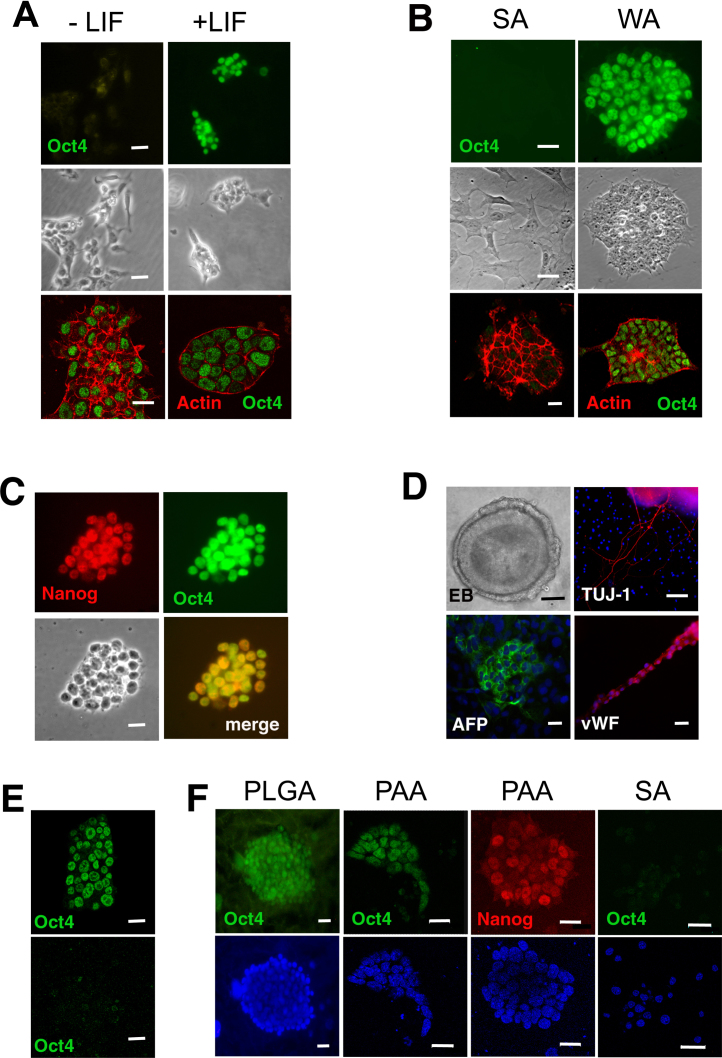
The effect of culture substrates on mESC self-renewal and pluripotency. (A) Top and middle panels: Oct-4 immunofluorescence (green) and phase contrast photomicrographs of E14 ESCs, 4 days after culture in the absence and presence of 1000 U/ml LIF on strong adhesive substrates (dishes coated with gelatin and 10% FBS). Bottom panels: F-actin (red) and Oct-4 immunofluorescence (green) in E14 ESCs 2 days following culture without and with LIF. Note cytoskeletal re-arrangement prior to loss of Oct-4. (B) Top and middle panels show Oct-4 immunofluorescence and phase contrast of E14 ESCs without LIF on dishes coated with gelatin/10% FBS (strong adhesion, SA) or 2.5% FBS (weak adhesion, WA) after 8 days (3 passages). Bottom panels show dual Oct-4 and actin immunofluorescence after the 3 passages. (C) Confocal images of R1 ESCs cultured on WA substrate for 2 passages and immunostained for Nanog (red) and Oct-4 (green). (D) Day 5 cavitated E14 embryoid body derived from ESCs cultured on WA substratum without LIF for 12 passages. The three remaining panels show immunofluorescence for TUJ1 (neuronal) AFP (endodermal) and von Willebrand Factor vWF (mesodermal) markers in cells derived from the embryoid bodies. (E) Oct-4 immunofluorescence in LIFR^−/−^ ESCs cultured on WA (top panel) and SA (bottom panel) substrates for 4 days. (F) DAPI nuclear staining and Oct-4 and Nanog expression in E14 ESCs cultured without LIF for 4 weeks on poly(lactic-co-glycolic acid) (PLGA) or plasma polymerised acrylic acid (PAA) synthetic substrates and SA substrates. Scale bars: A, 20 μm; B, 15 μm, C, 15 μm; D, 20 μm; E, 15 μm; F, 20 μm. (For interpretation of the references to colour in this figure legend, the reader is referred to the web version of this article.)

E14 mESCs cultured on FBS-coated substrates in the presence of LIF, maintained expression of the ESC marker, Oct-4, and grew as unspread, round cells in small colonies where cell–cell contact was maintained, whereas without LIF, the cells lost Oct-4 and started to spread on the substratum, leading to reduced colony formation (Fig. 1A). In the initial stages of LIF withdrawal, although the majority of cells were still Oct-4 positive, changes were observed in the actin cytoskeleton; thus, instead of being restricted to the outer membrane of cells at the periphery of ESC colonies, cortical F-actin was detected around the entire membrane of all cells after two days culture without LIF (Fig. 1A). Because the changes in cell morphology and actin redistribution occurred prior to loss of Oct-4 expression, we investigated if cell spreading itself affects self-renewal. To do this, we first reduced the adhesivity of tissue culture substrates by omitting gelatine and decreasing the FBS concentration used for coating the substratum from 10% to 2.5%. After 3 passages on the weakly adhesive substratum, E14 ESCs continued to express Oct-4 even in the absence of LIF, and cortical actin remained near the outer surfaces of cells located at the colony periphery. In contrast, Oct 4 could no longer be detected in cells cultured for the same time period on strongly adhesive substrates in the absence of LIF (Fig. 1B). Similarly, the R1 mESC line maintained expression of markers of self-renewal when cultured on the weakly adhesive substratum without LIF, indicating that this LIF-independent self-renewal is not restricted to one mESC line (Fig. 1C). Furthermore, following culture on weakly adhesive substrates for at least 12 passages in the absence of LIF, mESCs were still able to differentiate as cavitating embryoid bodies with well-defined ectodermal and endodermal epithelia, the cells of which went on to express markers for derivatives of all 3 germ layers (Fig. 1D). On the other hand, mESCs cultured on strongly adhesive substrates in the absence of LIF could not self-renew beyond the third passage. Although the increase in mESC numbers on weakly adhesive substrates was lower in the absence of LIF (Supplementary Fig. 1), in agreement with previous work showing that LIF promotes mESC proliferation (Viswanathan et al., 2003), the results demonstrate that self-renewing mESCs remain pluripotent during prolonged culture on the weakly adhesive substratum in the absence of LIF.

Supplementary Fig. I

To exclude the possibility that any endogenous LIF production by the ESCs might have helped maintain their undifferentiated state (Robertson et al., 1993), we demonstrated that LIFR^−/−^ mESCs also maintained Oct-4 expression on the weakly adhesive substratum (Fig. 1E) even though they are unable to respond to LIF (Dani et al., 1998). Additionally, to exclude the possibility that the observed effects on mESC self-renewal were due to the fact that the substrates had been coated with serum, which might contain growth factors or cytokines, we investigated the ability of two chemically distinct synthetic culture substrates to maintain the self-renewal of the E14 and R1 mESC lines. Synthetic substrates consisting of either poly(lactic-co-glycolic acid) (PLGA) or plasma polymerised acrylic acid (pPAA) allowed both E14 (Fig. 1F) and R1 cells (not shown) to attach, but they did not spread on these substrates. Compact colonies expressing Oct-4 and Nanog could be maintained in the absence of LIF under serum-free conditions for at least 4 weeks/6 passages (Fig. 1F). Taken together, these observations show that mESC lines may be maintained in a self-renewing pluripotent state in the absence of LIF or serum components if they are maintained on weakly adhesive culture substrates (dishes coated with 2.5% FBS, PLGA or pPAA). It remains to be seen if the action of LIF is mediated by the changes it induces in cell spreading under differing culture conditions.

**Fig. 2:**
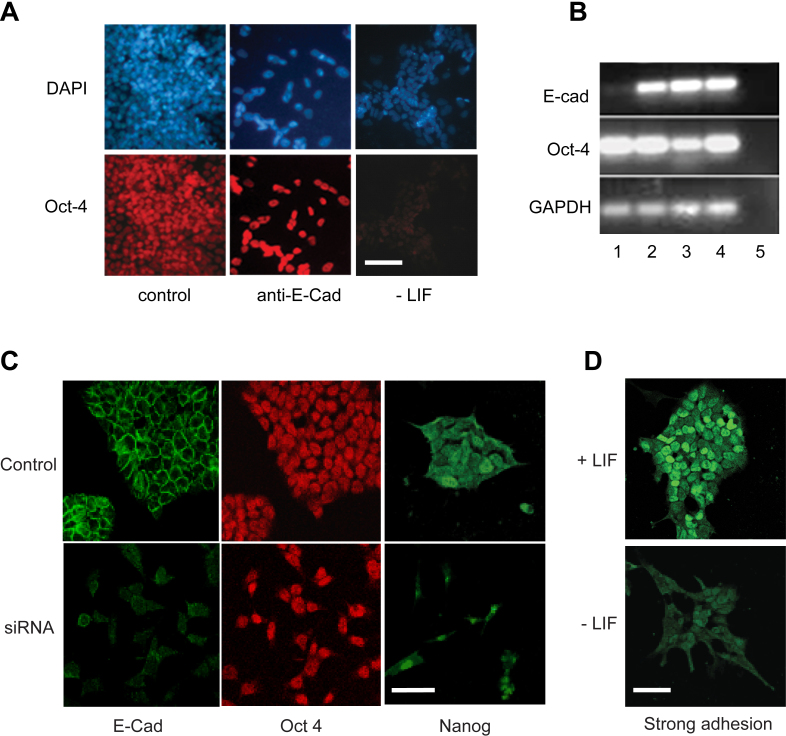
The role of E-cadherin in mESC self-renewal due to LIF and weakly adhesive substrata. (A) Photomicrographs of E14 ESCs cultured in the presence or absence of LIF on a strongly adhesive substrate for 5 days in the presence of 40 μg/ml rat anti-mouse E-cadherin (E-Cad) function-blocking antibody (Clone ECCD-1, Invitrogen) or control rat IgG. Note that while the cells did not grow in large colonies in the presence of the anti-E-Cad antibodies, Oct-4 (red fluorescence) was maintained (blue, DAPI). In contrast, Oct-4 was lost from the cells grown in the absence of LIF during this time period. (B) RT-PCR analysis of E14 ESCs cultured for 48 h in the presence of E-Cad-targeting siRNA (lane 1), non-targeting siRNA (lane 2), lipofectamine (lane 3), standard culture conditions (lane 4). Lane 5 is a no-template control. (C) E14 mESCs cultured for 2 days in the presence of E-cadherin-targeting or non-targeting siRNA (control) on a weakly adhesive substrate. Note that E-cadherin siRNA reduced E-cadherin immunofluorescence and colony formation, while Oct-4 and Nanog immunofluorescence was maintained. (D) Nanog immunoreactivity decreased in the absence of LIF on the strongly adhesive substrate after 2 days culture. Scale bars: 20 μm. (For interpretation of the references to colour in this figure legend, the reader is referred to the web version of this article.)

Given that unspread ESCs tended to grow in compact colonies, we investigated if the increased intercellular contact in colonies seen in response to LIF or the weakly adhesive substrates (dishes coated with 2.5% FBS) was required for ESC self-renewal. To this end, E14 mESC intercellular adhesion was inhibited with E-cadherin function-blocking antibodies (Larue et al., 1996) and siRNA knock-down. The results show that Oct-4 expression was maintained in the single cells resulting from anti-E-cadherin blockade of cell–cell adhesion on a strongly adhesive substratum (coated with gelatin and 10% FBS) in the presence of LIF, whereas in the absence of LIF the mESCs lost Oct4 expression by this time (Fig. 2A). Thus, Oct-4 expression is not dependent on the E-cadherin within mESC colonies resulting from culture in the presence of LIF. To determine if maintenance of mESC self-renewal on weakly adhesive substrates (dished coated with 2.5% FBS) in the absence of LIF was also independent of E-cadherin-mediated cellular interactions, E-cadherin siRNA was used to knockdown E-cadherin mRNA levels (Fig. 2B). Significantly, the immunoreactivities of both Oct4 and Nanog were maintained despite the loss of E-cadherin (Fig. 2C), although on a strongly adhesive substrate (coated with gelatin and 10% FBS), Nanog immunoreactivity was markedly reduced at this timepoint in the absence of LIF (Fig. 2D). Taken together, these observations indicate that changes in E-cadherin-mediated intercellular adhesion by either LIF or weakly adhesive substrates (coated with 2.5% FBS) do not regulate mESC self-renewal, and therefore the differentiation of mESCs is likely to result from increased cell–substratum adhesion and/or consequent shape changes per se. In support of this, a previous report has shown that absence of E-cadherin promotes mESC self-renewal in response to activin and nodal signalling (Soncin et al., 2009). The demonstration that blockade of activin signalling not only promoted differentiation but also dramatically increased cell spreading in that report (Soncin et al., 2009), is consistent with our hypothesis that the degree of cell–substratum adhesion plays a crucial role in the regulation of mESC self-renewal.

**Fig. 3:**
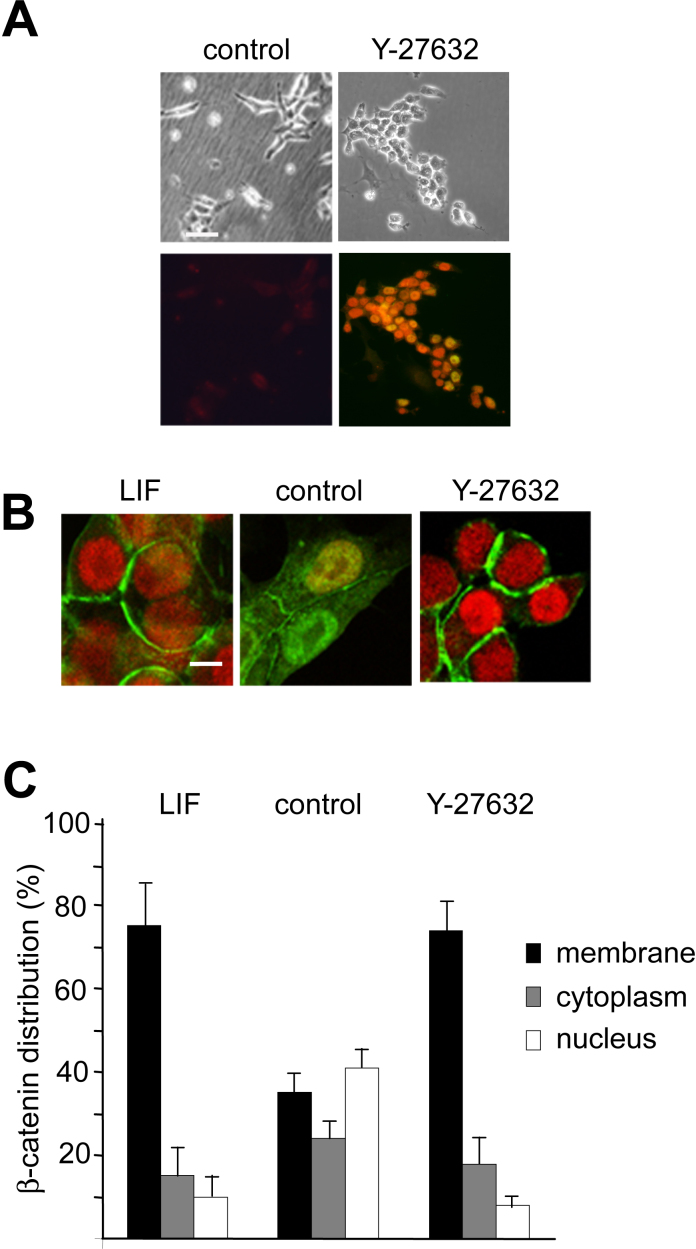
Effects of Rho kinase inhibition on ES cell self-renewal and β-catenin distribution. (A) E14 ESCs after 10 passages without LIF on a gelatine/10% (v/v) FBS-coated substratum in the absence (control) or presence of 10 μM Y-27632 Rho kinase (ROCK) inhibitor. The immunofluorescence shows merged Oct-4 (red) and Nanog (green) signals. (B) Confocal image showing immunofluorescence staining for β-catenin (green) and Oct-4 (red). Note that in the absence of LIF and the ROCK inhibitor, most of the β-catenin is in the nuclei, whereas in the presence of either Y-27632 or LIF, the signal for β-catenin is low in the Oct-4-positive nuclei but high in the cell membrane. (C) Mean percentage of total cellular β-catenin immunoreactivity in cell membrane, cytoplasmic and nuclear compartments. Error bars represent standard deviations; *n* = 10. Levels in each compartment are significantly different between the control group compared to those of the +LIF and Y-27632 groups (*P* < 0.001; unpaired Student's *t*-test). There is no significant difference between β-catenin levels between compartments of the +LIF and +Y-27632 groups (*P* > 0.5; unpaired Student's *t*-test). Scale bars: (A) 25 μm; (B) 2 μm. (For interpretation of the references to colour in this figure legend, the reader is referred to the web version of this article.)

Because adhesion-mediated mESC self-renewal involved dramatic changes in the actin cytoskeleton (Fig. 1), we investigated if direct manipulation of the cytoskeleton could itself affect ESC self-renewal. The Y-27632 Rho kinase (ROCK) inhibitor has been shown to reduce cell spreading by disruption of the actin cytoskeleton (Maekawa et al., 1999; McBeath et al., 2004), and is non-toxic for ESCs or their derivatives (Li et al., 2004; Watanabe et al., 2007). Significantly, the ROCK inhibitor not only inhibited mESC spreading and promoted colony formation, but also resulted in maintenance of Oct-4 and Nanog expression in the absence of LIF (Fig. 3A), although mESC numbers were lower than when cultured in the presence of LIF (Supplementary Fig. 1), consistent with lower proliferation or survival (Viswanathan et al., 2003). Quantitative PCR demonstrated that both the weakly adhesive substrate (dishes coated with 2.5% FBS) and the ROCK inhibitor resulted in increased levels of Oct-4 and Nanog mRNA in ESCs cultured in the absence of LIF (Supplementary Fig. 2). Furthermore, we found that Oct-4 levels did not differ between cells cultured in the presence of LIF and those cultured in the absence of LIF on either weakly adhesive substrates (dishes coated with 2.5% FBS) or in the presence of ROCK inhibitor. Interestingly, the ROCK inhibitor can prevent the differentiation of human ESCs cultured in the absence of feeder cells (Pakzad et al., 2010; Kitajima and Niwa, 2010), indicating that the ability of the ROCK inhibitor to promote self-renewal is not restricted to mESCs. Taken together, the above experiments indicate that reduced cell spreading, either by manipulation of cell–substratum adhesivity or by inhibition of Rho kinase, promotes ESC self-renewal.

Supplementary Fig. II

To establish the relationships between mESC self-renewal mediated by LIF and cell shape changes caused by the inhibition of ROCK, or manipulations of the culture substratum, we next investigated involvement of the β-catenin and STAT3 intracellular signalling pathways previously implicated in self-renewal (Sato et al., 2004; Hao et al., 2006; Burdon et al., 2002). Confocal microscopy demonstrated that most β-catenin was localised to the cell membrane in Oct-4-positive ESCs maintained with either LIF or the ROCK inhibitor (Fig. 3B and C). In contrast, in the absence of either the ROCK inhibitor or LIF, β-catenin was mainly present in the nucleus of differentiating ESCs displaying reduced Oct-4 expression (Fig. 3B and C). These observations indicate that nuclear β-catenin is not involved in the promotion of ESC self-renewal mediated by the ROCK inhibitor or LIF, and are consistent with previous evidence demonstrating a dramatic increase in β-catenin-mediated transcription on differentiation (Otero et al., 2004; Dravid et al., 2005), and a recent study showing that Wnt/β-catenin signalling promotes the differentiation of human ESCs (Davidson et al., 2012).

**Fig. 4:**
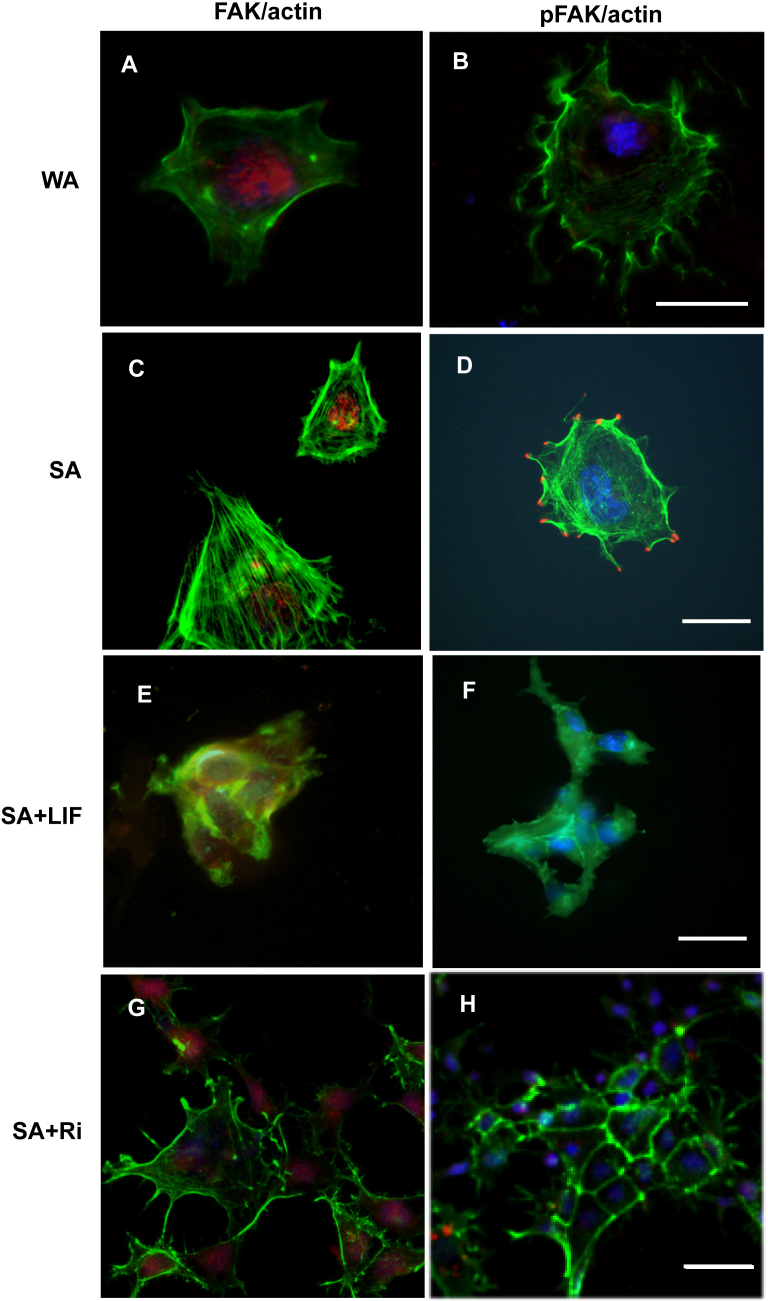
The effects of LIF and Rho kinase inhibition on actin stress fibre and focal adhesion formation in mESCs cultured on weakly and strongly adhesive substrata. Fluorescence images showing actin (green phalloidin staining) and FAK (A, C, E and G) or pFAK red immunofluorescence, and blue DAPI counterstaining (B, D, F and H). A and B: weakly adhesive substrates (dishes coated 2.5% FBS); C–H: strongly adhesive substrates (dishes coated with gelatin and 10% FBS); E and F: cultures supplemented with 1000 U/ml LIF; G and H: cultures supplemented with 10 μM Y-27632 Rho kinase (ROCK) inhibitor. Cultures were fixed and processed for immunomicroscopy after 4 days of culture. Scale bars 20 μm. Note that low FAK immunoreactivity is present in the vicinity of cell nuclei under all conditions (A, C, E and G), whereas pFAK-positive peripheral focal contacts (D) and actin stress-fibres (C and D) are only present on the strong adhesive substrate where the cells spread in the absence of LIF. (For interpretation of the references to colour in this figure legend, the reader is referred to the web version of this article.)

It has recently been shown that mESC self-renewal in the presence of LIF is critically dependent on the degree of cell spreading (Hayashi et al., 2007; Hunt et al., 2012). Thus, increased cell spreading caused by strongly adhesive substrates (produced using large amounts of fibronectin) results in mESC differentiation (Hayashi et al., 2007) together with increased levels of phosphorylated focal adhesion kinase (pFAK) and the appearance of focal adhesions around the periphery of highly spread cells (Hunt et al., 2012). To investigate the relationship between LIF- and spreading-induced mESC differentiation, we demonstrate here that on the weakly adhesive substrate that permits LIF-independent mESC self-renewal (Fig. 1B), there are no pFAK-positive focal adhesions or actin stress-fibres, actin instead being accumulated around the periphery of the cell (Fig. 4A and B). In contrast, on the strongly adhesive substrates where LIF is necessary to prevent differentiation (Fig. 1A), the LIF not only reduces cell spreading, but also inhibits formation of actin stress-fibres and there are no peripheral focal adhesions as displayed by the presence of pFAK (Fig. 4C–F). Similarly, there are no pFAK-positive focal adhesions or actin stress-fibres on strongly adhesive substrates after ROCK inhibition (Fig. 4G and H) which also results in mESC self-renewal (Fig. 3). These results extend the previous reports (Hayashi et al., 2007, Hunt et al., 2012) by showing that substrate adhesivity and LIF are functionally interchangeable in their regulation of mESC spreading, stress-fibre and focal contact formation, which parallels their abilities to regulate mESC differentiation.

**Fig. 5:**
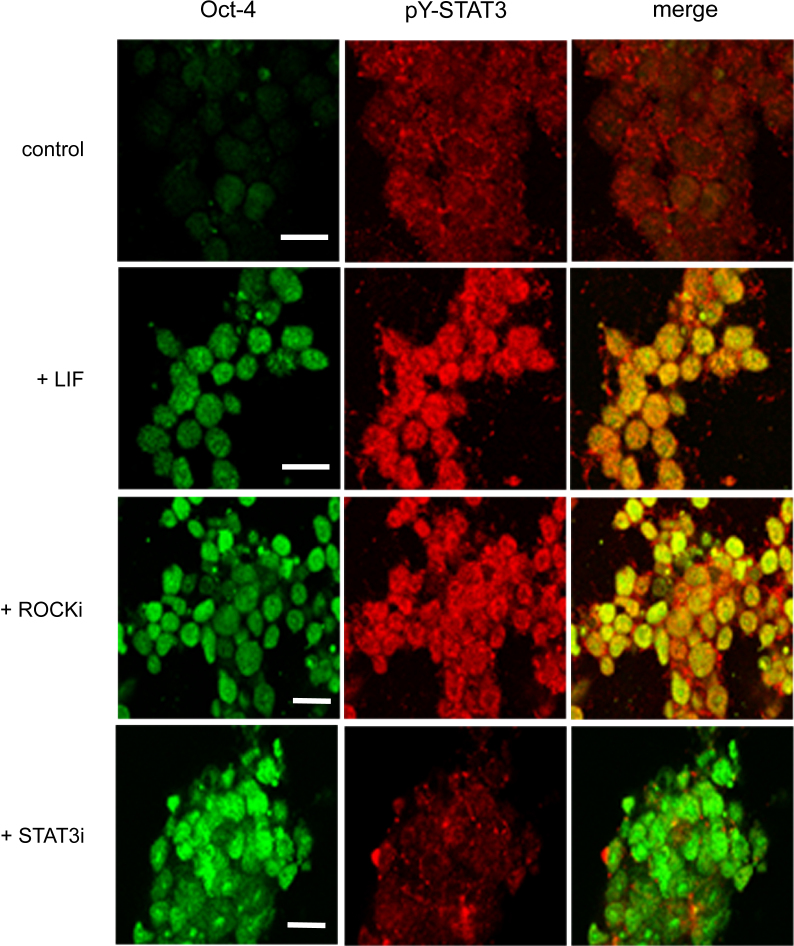
The relationship between mESC self-renewal and phosphorylated STAT3. Confocal images showing Oct-4 (red), pY-STAT3 (green) and merged expression in E14 ESCs 4 days following third re-plating on strong adhesive substrate. The additions of LIF (1000 U/ml), Y-27632 Rho kinase inhibitor (10 μM) and the STAT3 inhibitor (100 μM) are indicated. Nuclear pY-STAT3 is readily detectable in the nuclei of Oct-4-positive cells in the presence of LIF or Y-27632 but absent in Oct-4-negative controls (top panel). In contrast, there is little pY-STAT3 immunoreactivity in the nuclei of cells treated with the STAT3 inhibitor, although they maintain Oct-4 levels in the presence of the Y-27632 Rho kinase inhibitor. Scale bars: 15 μm. (For interpretation of the references to colour in this figure legend, the reader is referred to the web version of this article.)

High levels of activated tyrosine-phosphorylated STAT3 (pY-STAT3) were observed in the nuclei of ESCs cultured in the presence of either LIF or the ROCK inhibitor, but in the absence of these agents the cells displayed low levels of nuclear pY-STAT3, and as expected, differentiated, evidenced by the lack of Oct-4 (Fig. 5). When the cells were cultured with a cell-permeable STAT3 inhibitor peptide (STAT3i, Calbiochem), which blocks STAT3 tyrosine phosphorylation and nuclear translocation (Turkson et al., 2001), nuclear pY-STAT3 was lost (Fig. 5). Significantly, the ESCs remained Oct-4 positive if the ROCK inhibitor was present, despite the absence of nuclear pY-STAT3 (Fig. 5). These observations indicate that STAT3 tyrosine phosphorylation and nuclear translocation are not absolute requirements for ESC self-renewal. This conclusion is supported by a report indicating that STAT3 is not required for self-renewal if ESCs are cultured in the presence of an ERK inhibitor (Ying et al., 2008). Moreover, a recent study has shown that if mESCs cultured in the presence of LIF are prevented from adhering to a substratum, forcing them to form embryoid bodies, then the mESCs differentiate, despite the fact that pY-STAT3 is expressed (Hunt et al., 2012). Taken together with our own findings, these studies suggest that the effect of STAT3 on mESC self-renewal is dependent on the culture conditions used.

There are a number of potential mechanisms whereby cell spreading could determine mESC fate, despite the lack of involvement of the known signalling pathways mediated by nuclear translocation of β-catenin and pY-STAT3. For example, the ability of cell morphology to regulate differentiation has been shown to involve the generation of cytoskeletal tension via RhoA/ROCK (Huang and Ingber, 2000; McBeath et al., 2004). Significantly, cytoskeletal tension can in turn affect the localisation (and hence activity) of signalling pathway components, including ERK (Aplin and Juliano, 2001) and Akt (Sawada and Sheetz, 2002), known to promote the differentiation of mESCs (Burdon et al., 1999; Chen et al., 2000; Ying et al., 2008). In the light of the observations we report here, it is likely that cell shape and cytoskeletal tension exert similar regulatory roles in mESCs and may be the common pathway by which both LIF and cell–substratum adhesion regulate mESC differentiation.

## References

[bib0005] Anneren C., Cowan C.A., Melton D.A. (2004). The Src family of tyrosine kinases is important for embryonic stem cell self-renewal. J Biol Chem.

[bib0010] Aplin A.E., Juliano R.L. (2001). Regulation of nucleocytoplasmic trafficking by cell adhesion receptors and the cytoskeleton. J Cell Biol.

[bib0015] Burdon T., Chambers I., Stracey C., Niwa H., Smith A. (1999). Signaling mechanisms regulating self-renewal and differentiation of pluripotent embryonic stem cells. Cells Tissues Organs.

[bib0020] Burdon T., Smith A., Savatier P. (2002). Signalling, cell cycle and pluripotency in embryonic stem cells. Trends Cell Biol.

[bib0025] Chen T.T., Yuan D.T., Wei B., Jiang J., Kang J.H., Ling K. (2010). E-cadherin-mediated cell–cell contact is critical for induced pluripotent stem cell generation. Stem Cells.

[bib0030] Chowdhury F., Na S., Li D., Poh Y.C., Tanaka T.S., Wang F. (2010). Material properties of the cell dictate stress-induced spreading and differentiation in embryonic stem cells. Nat Mater.

[bib0035] Connelly J.T., Gautrot J.E., Trappmann B., Tan D.W.M., Donati G., Huck W.T.S. (2010). Actin and serum response factor transduce physical cues from the microenvironment to regulate epidermal stem cell fate decisions. Nat Cell Biol.

[bib0040] Costa P., Almeida F.V.M., Connelly J.T. (2012). Biophysical signals controlling cell fate decisions: how do stem cells really feel. Int J Biochem Cell B.

[bib0045] Chen Y., Li X., Eswarakumar V.P., Seger R., Lonai P. (2000). Fibroblast growth factor (FGF) signaling through PI 3-kinase and Akt/PKB is required for embryoid body differentiation. Oncogene.

[bib0050] Dani C., Chambers I., Johnstone S., Robertson M., Ebrahimi B., Saito M. (1998). Paracrine induction of stem cell renewal by LIF-deficient cells: a new ES cell regulatory pathway. Dev Biol.

[bib0055] Davidson K.C., Adams A.M., Goodson J.M., McDonald C.E., Potter J.C., Berndt J.D. (2012). Wnt/beta-catenin signaling promotes differentiation, not self-renewal, of human embryonic stem cells and is repressed by Oct4. Proc Natl Acad Sci USA.

[bib0060] Dravid G., Ye Z., Hammond H., Chen G., Pyle A., Donovan P. (2005). Defining the role of Wnt/beta-catenin signaling in the survival, proliferation, and self-renewal of human embryonic stem cells. Stem Cells.

[bib0065] France R.M., Short R.D., Dawson R.A., MacNeil S. (1998). Attachment of human keratinocytes to plasma co-polymers of acrylic acid/octa-1,7-diene and allyl amine/octa-1,7-diene. J Mater Chem.

[bib0070] Fuchs E., Tumbar T., Guasch G. (2004). Socializing with the neighbors: stem cells and their niche. Cell.

[bib0075] Gao L., McBeath R., Chen C.S. (2010). Stem cell shape regulates a chondrogenic versus myogenic fate through Rac1 and N-cadherin. Stem Cells.

[bib0080] Hao J., Li T.G., Qi X., Zhao D.F., Zhao G.Q. (2006). WNT/beta-catenin pathway up-regulates Stat3 and converges on LIF to prevent differentiation of mouse embryonic stem cells. Dev Biol.

[bib0085] Hayashi Y., Furue M.K., Okamoto T., Ohnuma K., Myoishi Y., Fukuhara Y. (2007). Integrins regulate mouse embryonic stem cell self-renewal. Stem Cells.

[bib0090] Huang S., Ingber D.E. (2000). Shape-dependent control of cell growth, differentiation, and apoptosis: switching between attractors in cell regulatory networks. Exp Cell Res.

[bib0095] Hunt G.C., Singh P., Schwarzbauer J.E. (2012). Endogenous production of fibronectin is required for self-renewal of cultured mouse embryonic stem cells. Exp Cell Res.

[bib0100] Kitajima H., Niwa H. (2010). Clonal expansion of human pluripotent stem cells on gelatin-coated surfaces. Biochem Biophys Res Commun.

[bib0105] Larue L., Antos C., Butz S., Huber O., Delmas V., Dominis M. (1996). A role for cadherins in tissue formation. Development.

[bib0110] Li L., Arman E., Ekblom P., Edgar D., Murray P., Distinct Lonai P. (2004). GATA6- and laminin-dependent mechanisms regulate endodermal and ectodermal embryonic stem cell fates. Development.

[bib0115] McBeath R., Pirone D.M., Nelson C.M., Bhadriraju K., Chen C.S. (2004). Cell shape, cytoskeletal tension, and RhoA regulate stem cell lineage commitment. Dev Cell.

[bib0120] Maekawa M., Ishizaki T., Boku S., Watanabe N., Fujita A., Iwamatsu A. (1999). Signaling from Rho to the actin cytoskeleton through protein kinases ROCK and LIM-kinase. Science.

[bib0125] Murray P., Hayward S.A., Govan G.G., Gracey A.Y., Cossins A.R. (2007). An explicit test of the phospholipid saturation hypothesis of acquired cold tolerance in *Caenorhabditis elegans*. Proc Natl Acad Sci USA.

[bib0130] Nichols J., Chambers I., Taga T., Smith A. (2001). Physiological rationale for responsiveness of mouse embryonic stem cells to gp130 cytokines. Development.

[bib0135] Otero J.J., Fu W., Kan L., Cuadra A.E., Kessler J.A. (2004). Beta-catenin signaling is required for neural differentiation of embryonic stem cells. Development.

[bib0140] Pakzad M., Totonchi M., Taei A., Seifinejad A., Hassani S.N., Baharvand H. (2010). Presence of a rock inhibitor in extracellular matrix supports more undifferentiated growth of feeder-free human embryonic and induced pluripotent stem cells upon passaging. Stem Cell Rev Rep.

[bib0145] Redmer T., Diecke S., Grigoryan T., Quiroga-Negreira A., Birchmeier W., Besser D. (2011). E-cadherin is crucial for embryonic stem cell pluripotency and can replace OCT4 during somatic cell reprogramming. EMBO Reports.

[bib0150] Robertson M., Chambers I., Rathjen P., Nichols J., Smith A. (1993). Expression of alternative forms of differentiation inhibiting activity (DIA/LIF) during murine embryogenesis and in neonatal and adult tissues. Dev Genet.

[bib0155] Sato N., Meijer L., Skaltsounis L., Greengard P., Brivanlou A.H. (2004). Maintenance of pluripotency in human and mouse embryonic stem cells through activation of Wnt signaling by a pharmacological GSK-3-specific inhibitor. Nat Med.

[bib0160] Soncin F., Mohamet L., Eckardt D., Ritson S., Eastham A., Bobola N. (2009). Abrogation of E-cadherin mediated cell–cell contact in mouse embryonic stem cells results in reversible LIF-independent self-renewal. Stem Cells.

[bib0165] Sawada Y., Sheetz M.P. (2002). Force transduction by Triton cytoskeletons. J Cell Biol.

[bib0170] Smyth N., Vatansever H.S., Murray P., Frie C., Paulsson M., Edgar D. (1999). Absence of basement membranes after targeting the *LAMC1* gene results in early embryonic lethality due to failure of endoderm differentiation. J Cell Biol.

[bib0175] Turkson J., Ryan D., Kim J.S., Zhang Y., Chen Z., Haura E. (2001). Phosphotyrosyl peptides block Stat3-mediated DNA binding activity, gene regulation, and cell transformation. J Biol Chem.

[bib0180] Uda Y., Poh Y.C., Chowdhury F., Wu D.C., Tanaka T.S., Satob M. (2011). Force via integrins but not E-cadherin decreases Oct3/4 expression in embryonic stem cells. Biochem Biophys Res Commun.

[bib0185] Viswanathan S., Benatar T., Mileikovsky M., Lauffenburger D.A., Nagy A., Zandstra P.W. (2003). Supplementation-dependent differences in the rates of embryonic stem cell self-renewal, differentiation, and apoptosis. Biotechnol Bioeng.

[bib0190] Watanabe K., Ueno M., Kamiya D., Nishiyama A., Matsumura M., Wataya T. (2007). A ROCK inhibitor permits survival of dissociated human embryonic stem cells. Nat Biotechnol.

[bib0195] Wells N., Baxter M.A., Turnbull J.E., Murray P., Edgar D., Parry K.L. (2009). The geometric control of E14 and R1 mouse embryonic stem cell pluripotency by plasma polymer surface chemical gradients. Biomaterials.

[bib0200] Ying Q-L., Wray J., Nichols J., Batlle-Morera L., Doble B., Woodgett J. (2008). The ground state of embryonic stem cell self-renewal. Nature.

[bib0205] Zhang H., Hussain I., Brust M., Butler M.F., Rannard S.P., Cooper A.I. (2005). Aligned two- and three-dimensional structures by directional freezing of polymers and nanoparticles. Nat Mater.

