# Supplementary material for: The self-renewal of mouse embryonic stem cells is regulated by cell–substratum adhesion and cell spreading
Source: Int J Biochem Cell Biol. 2013 Nov;45(11):2698–705. doi: 10.1016/j.biocel.2013.07.001 (PMC3898852; doi:10.1016/j.biocel.2013.07.001)
Supplement: Supplementary Fig. I [file mmc1.ppt]

## Slide 1
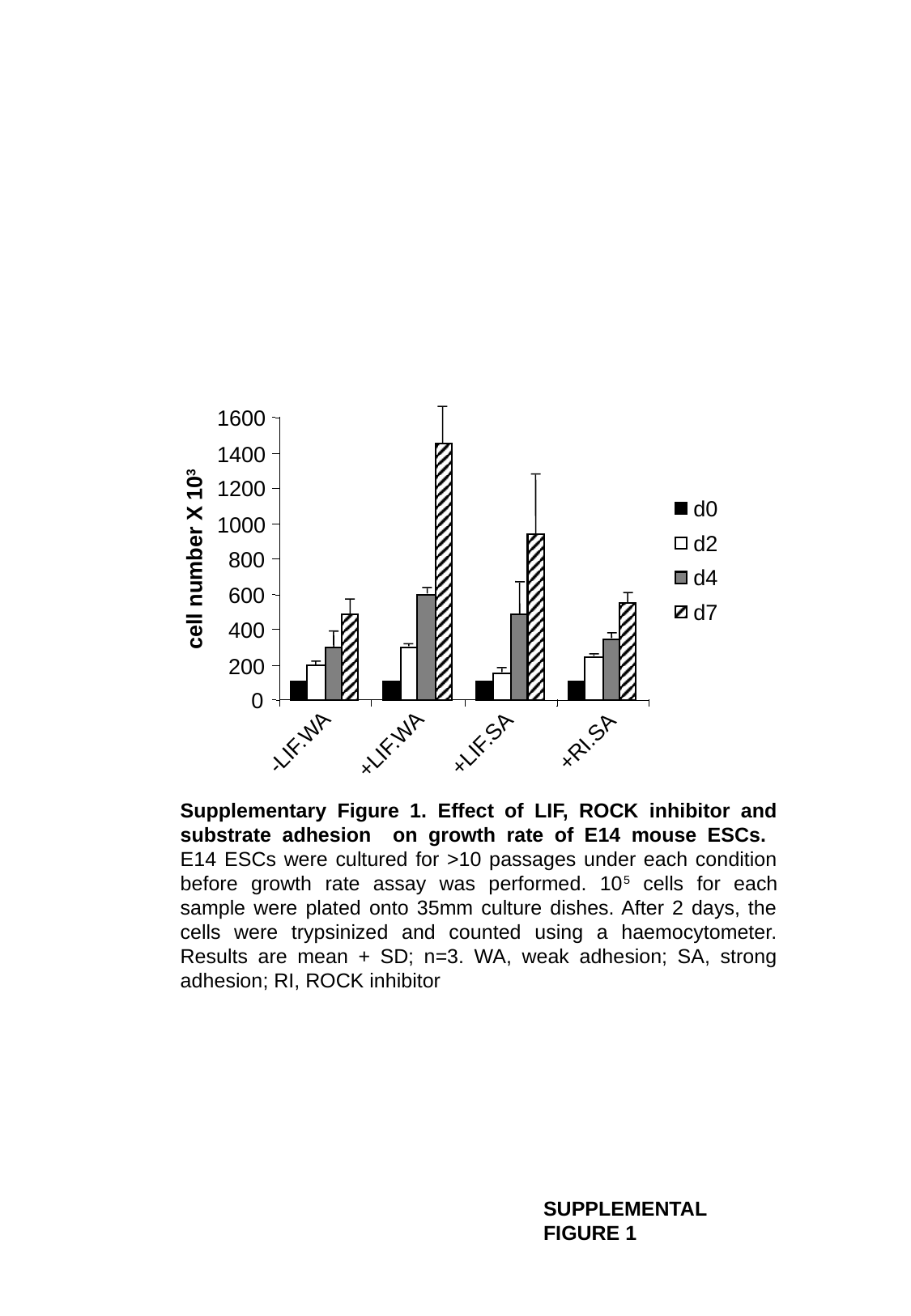

1600
1400
1200
d0
1000
800
600
400
d2
cell number X 103
d4
d7
200
0
+RI.SA
-LIF.WA
+LIF.SA
+LIF.WA
Supplementary Figure 1. Effect of LIF, ROCK inhibitor and substrate adhesion on growth rate of E14 mouse ESCs.
E14 ESCs were cultured for >10 passages under each condition before growth rate assay was performed. 105 cells for each sample were plated onto 35mm culture dishes. After 2 days, the cells were trypsinized and counted using a haemocytometer. Results are mean + SD; n=3. WA, weak adhesion; SA, strong adhesion; RI, ROCK inhibitor
SUPPLEMENTAL FIGURE 1
