# Supplementary material for: The self-renewal of mouse embryonic stem cells is regulated by cell–substratum adhesion and cell spreading
Source: Int J Biochem Cell Biol. 2013 Nov;45(11):2698–705. doi: 10.1016/j.biocel.2013.07.001 (PMC3898852; doi:10.1016/j.biocel.2013.07.001)
Supplement: Supplementary Fig. II [file mmc2.pptx]

## Slide 1
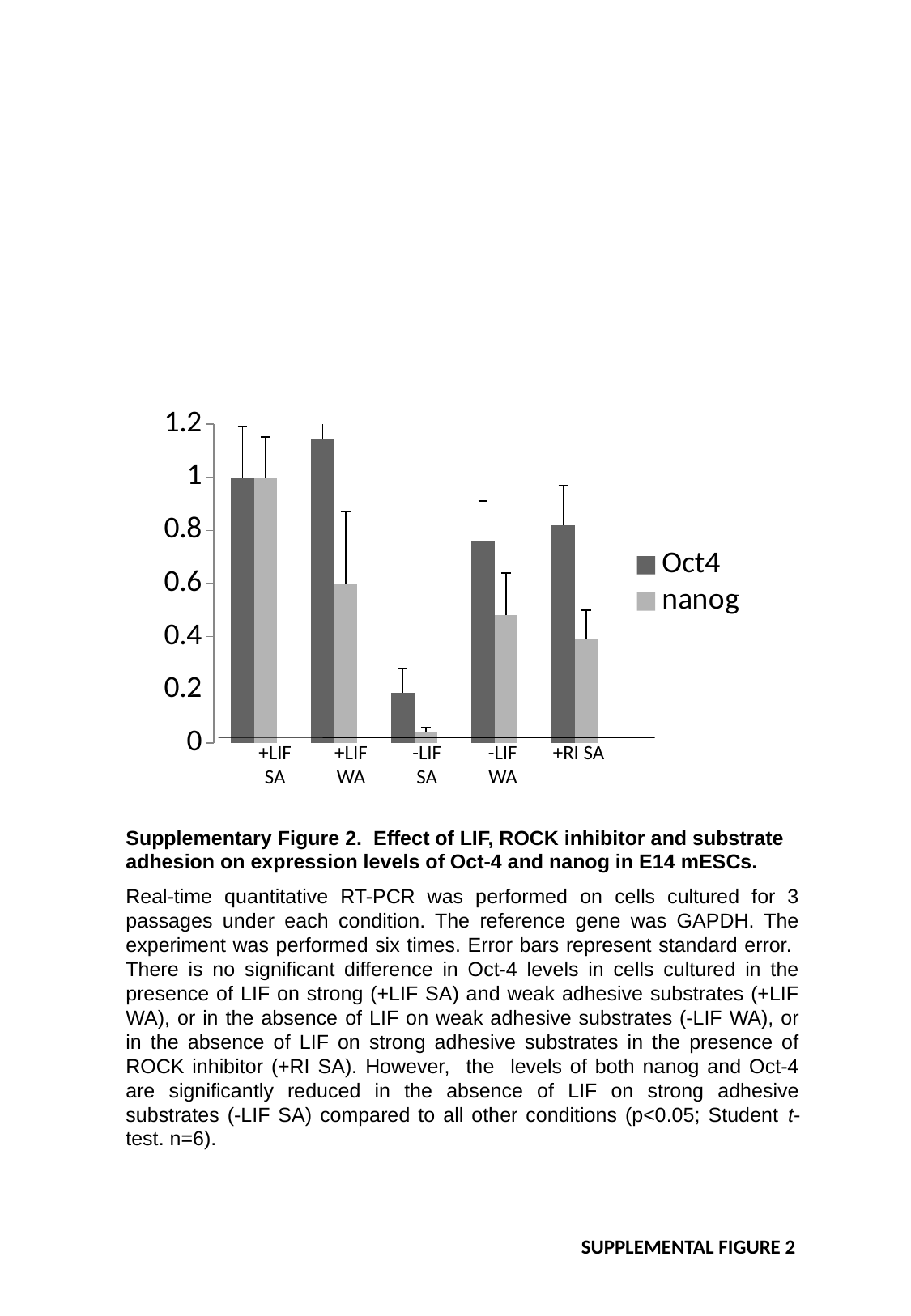

### Chart
| Category | | |
|---|---|---|+LIF SA
+LIF WA
-LIF SA
-LIF WA
+RI SA
Supplementary Figure 2. Effect of LIF, ROCK inhibitor and substrate adhesion on expression levels of Oct-4 and nanog in E14 mESCs.
Real-time quantitative RT-PCR was performed on cells cultured for 3 passages under each condition. The reference gene was GAPDH. The experiment was performed six times. Error bars represent standard error. There is no significant difference in Oct-4 levels in cells cultured in the presence of LIF on strong (+LIF SA) and weak adhesive substrates (+LIF WA), or in the absence of LIF on weak adhesive substrates (-LIF WA), or in the absence of LIF on strong adhesive substrates in the presence of ROCK inhibitor (+RI SA). However, the levels of both nanog and Oct-4 are significantly reduced in the absence of LIF on strong adhesive substrates (-LIF SA) compared to all other conditions (p<0.05; Student t-test. n=6).
supplemental figure 2
